# Increasing continuous positive airway pressure use rates in the delivery room for very preterm infants: a quality improvement initiative

**DOI:** 10.3389/fped.2025.1697565

**Published:** 2025-11-06

**Authors:** Hanna Kim, Na Hyun Lee, Hyeseon Kim, Seung Hyun Kim, Soo Jeong Choo, Misun Yang, So Yoon Ahn, Se In Sung, Yun Sil Chang

**Affiliations:** 1 Department of Pediatrics, Samsung Medical Center, Sungkyunkwan University School of Medicine, Seoul, Republic of Korea; 2 Cell and Gene Therapy Institute for Future Medicine, Samsung Medical Center, Seoul, Republic of Korea; 3 Department of Health Science and Technology, Samsung Advanced Institute for Health Sciences & Technology (SAIHST), Sungkyunkwan University, Seoul, Republic of Korea

**Keywords:** continuous positive airway pressure, bronchopulmonary dysplasia, intratrachealintubation, neonatal resuscitation, premature, quality improvement

## Abstract

**Background:**

In spontaneously breathing preterm infants, less invasive strategies, such as continuous positive airway pressure (CPAP) and less invasive surfactant administration, have been increasingly implemented to reduce lung injury. In 2018, our center initiated a noninvasive neonatal resuscitation protocol incorporating these approaches as quality improvement (QI) initiatives. We aimed to evaluate the feasibility, safety, and effectiveness of this protocol by comparing respiratory outcomes before and after its implementation.

**Methods:**

We retrospectively reviewed the medical records of 578 infants born at 25 + 0 to 29 + 6 weeks of gestation between 2014 and 2022 at Samsung Medical Center. Infants born in 2018 and those with severe congenital anomalies, delivery room deaths, or outborn status were excluded. The study population was divided into Period 1 (2014–2017, before noninvasive protocol implementation) and Period 2 (2019–2022, after protocol implementation) to assess the impact of QI initiatives on neonatal resuscitation practices. The year 2018 was excluded from the analysis as it represented a transitional period. We analyzed the rate of endotracheal intubation at birth and other respiratory outcomes, such as CPAP failure and bronchopulmonary dysplasia (BPD).

**Results:**

The rate of initial intubation was significantly lower in Period 2 than in Period 1 [77.0% vs. 45.9%; adjusted odds ratio (aOR), 0.24; 95% confidence interval (CI), 0.15–0.40; *P* < 0.001], with declines observed across all gestational groups, particularly among infants ≥26 weeks’ gestation. The rate of postnatal steroid use for BPD prevention was also significantly lower in Period 2 (50.0% vs. 15.3%, aOR, 0.12; 95% CI, 0.07–0.21; *P* < 0.001). In Period 2, among 68 infants initially managed with CPAP during Period 2, 15 (22.1%) experienced CPAP failure within 48 h, and 24 (35.3%) experienced failure at any time during hospitalization. Despite these failures, no significant differences in the incidence of BPD, duration of invasive ventilation, or mortality were observed between the two periods.

**Conclusions:**

Our findings suggest that even in extremely preterm infants, a noninvasive resuscitation strategy is feasible, associated with reduced postnatal steroid use, and does not worsen major neonatal outcomes, supporting its use as a viable alternative for those who do not require immediate intubation.

## 1 Introduction

Early application of continuous positive airway pressure (CPAP) in preterm infants can reduce the need for intubation and surfactant, but failure may lead to adverse events such as delayed intubation, respiratory acidosis, or air leak (1). With recent advances in noninvasive ventilation strategies, sustained noninvasive support has been shown to minimize exposure to invasive ventilation and postnatal steroids, and improves long-term outcomes (2–4). In line with this evidence, the 2015 Neonatal Resuscitation Program (NRP) guidelines recommend using CPAP, rather than undergoing routine intubation and surfactant administration, to support spontaneously breathing preterm infants with respiratory distress (5). In our institution, Samsung Medical Center, we have implemented a quality improvement (QI) initiative to increase CPAP use in delivery rooms since 2019. However, CPAP application in the delivery room for very preterm infants (VPI) remains challenging because of limited staff expertise, difficulty in maintaining support during transport, and a relatively high CPAP failure rate (6, 7). In Korea, these staffing-related challenges are particularly relevant even in high-resource settings, as outcomes of VPI still vary among NICUs, partly due to differences in neonatologist staffing (8). For context, Korean Neonatal Network data from 2019 to 2022, 65.8% of infants born at 25–29 weeks' gestation were intubated immediately after birth, compared to 45.9% at our institution during the same period (9). In this study, we aimed to assess the feasibility, safety, acceptability and effectiveness of the QI initiative in clinical practice by comparing two time periods before and after its implementation. In Period 1, 77% of infants underwent delivery room intubation, and about 41% of these were extubated within 24 h. Thus, approximately 45% of the total cohort remained intubated beyond 24 h, and we therefore pre-specified a primary target to reduce the delivery room intubation rate to ≤45% after implementation of the intervention.

## 2 Materials and methods

### 2.1 Study population

This retrospective cohort study was conducted at Samsung Medical Center, a tertiary neonatal intensive care unit (NICU) in Seoul, Korea. We reviewed the medical records of 578 very low birth weight infants born between 25 + 0 and 29 + 6 weeks' gestation from January 2014 to December 2022. Infants born in 2018, those with severe congenital anomalies, those who died in the delivery room, or those who were outborn were excluded.

The study population was divided into two groups based on the implementation of the QI initiative: Period 1 (January 2014 to December 2017) and Period 2 (January 2019 to December 2022). Infants were further stratified by gestational age into extremely preterm infants (EPI, <28 + 0 weeks) and VPI (≥28 + 0 weeks).

### 2.2 Initial respiratory strategy before and after QI

During Period 1, most infants born at 25–29 weeks of gestation underwent prophylactic intubation and surfactant administration immediately after birth. In the NICU, early extubation was performed if the infant was deemed clinically stable. Two or more healthcare providers participated in neonatal resuscitation, and there were no alternatives to intubation or free-flow oxygen for transport. Routine NRP training was conducted during this period.

In contrast, during Period 2, the revised protocol, emphasizing initial CPAP application in the delivery room for infants with spontaneous breathing and heart rates >100 bpm, was implemented. CPAP support was provided via a portable device (HAMILTON-T1, Hamilton Medical AG, Bonaduz, Switzerland) during transport to the NICU, followed by less invasive surfactant administration (LISA). If CPAP failed to provide adequate respiratory support, intubation was performed based on clinical judgment. To support this approach, resuscitation was conducted by three or more healthcare providers, including a skilled NICU nurse practitioner, and portable CPAP devices were always available in the delivery room. Routine NRP training was maintained, with additional hands-on LISA practice incorporated into staff education. The year 2018 was excluded from the analysis as it represented a transitional period during which portable CPAP and LISA were first introduced but not yet standardized.

### 2.3 Definition and data collection

We collected the following clinical data from the medical records of enrolled infants: perinatal demographic characteristics, such as gestational age, birth weight, multiple pregnancy, prolonged rupture of membranes (PROM; rupture lasting ≥24 h), as well as completion of antenatal steroid therapy and clinical outcomes. Completion was defined as administration of two doses of maternal betamethasone or dexamethasone, according to standard regimens, with delivery occurring at least 24 h after the final dose. Clinical outcomes were categorized as short-term, long-term, and other neonatal outcomes.

Short-term outcomes included 1) delivery room intubation status, 2) CPAP failure within 48 h, defined as the need for mechanical ventilation due to recurrent apnea requiring intervention, respiratory acidosis (pH < 7.2 and partial pressure of carbon dioxide >65 mmHg), or hypoxemia (fraction of inspired oxygen >0.4 to maintain peripheral oxygen saturation 90%–95%), 3) CPAP failure at any time during hospitalization, 4) surfactant administration (invasive or noninvasive), 5) air leak requiring chest tube insertion or needle aspiration; and 6) pulmonary hemorrhage, defined as blood in the airway or respiratory collapse.

Long-term outcomes included 1) bronchopulmonary dysplasia (BPD), defined according to the 2019 Jensen criteria as the requirement for supplemental oxygen or positive pressure respiratory support (invasive or noninvasive ventilation) at 36 weeks' postmenstrual age, with severity classified based on the mode of support; 2) late pulmonary hypertension, defined as echocardiographic evidence after 1 month of age, indicated by at least one of the following findings: a tricuspid regurgitant pressure gradient ≥40 mmHg, bidirectional/right-to-left shunting across the ductus arteriosus or foramen ovale, or interventricular septal flattening; 3) duration of invasive and noninvasive ventilation, defined as the cumulative number of days each infant received invasive or noninvasive respiratory support during entire NICU admission; and 4) postnatal steroid (dexamethasone) use for BPD prevention (10).

Other neonatal outcomes included: 1) intraventricular hemorrhage grade ≥3, classified according to Papile's criteria, 2) retinopathy of prematurity (ROP) requiring treatment, including laser photocoagulation or intravitreal anti-VEGF injection, 3) necrotizing enterocolitis stage ≥2, defined by the modified Bell's criteria, 4) culture-proven sepsis, confirmed by positive blood, cerebrospinal fluid, or urine cultures with clinical symptoms, and 5) mortality during hospitalization. Demographic factors and clinical outcomes were compared between Periods 1 and 2.

### 2.4 Statistical analysis

Categorical variables were compared using the chi-square test or Fisher's exact test, and continuous variables with Student's *t* test or the Mann–Whitney *U* test. Variables with *P* < 0.05 in univariate analysis were included in multivariate logistic regression models to identify independent associations with outcomes. Adjusted odds ratios (aORs) with 95% confidence intervals (CIs) were reported. All statistical analyses were performed using R version 4.3.1, and a *P* < 0.05 was considered statistically significant.

Data on initial intubation rates were collected quarterly, and statistical process control charts were constructed using Microsoft Excel to evaluate trends over time. The *x*-axis represented calendar quarters, and the *y*-axis indicated the proportion of infants intubated at birth. Upper and lower control limits were applied to detect significant process variations.

## 3 Results

**Figure 1:**
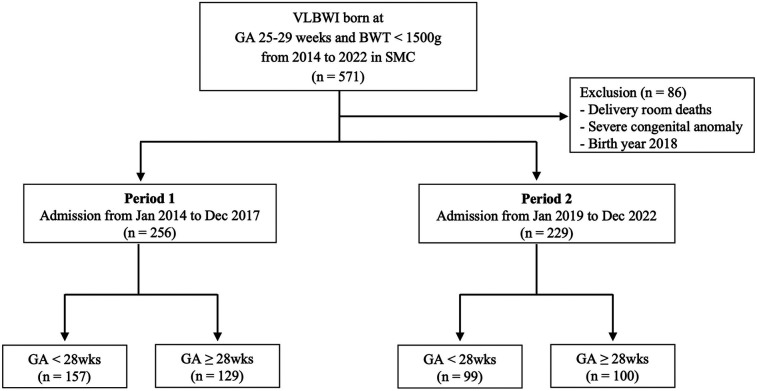
Flowchart of the study design for patient enrollment. VLBWI, very-low-birthweight infants; GA, gestational age; SMC, Samsung Medical Center.

A total of 578 infants born between 25 + 0 and 29 + 6 weeks’ gestation were initially screened. Upon excluding 89 infants born in 2018, 2 with severe congenital anomalies, and 2 who died in the delivery room, a total of 485 infants were included in the final analysis: 256 in Period 1 and 229 in Period 2 (Figure 1). The initial intubation rate was significantly lower in Period 2 than in Period 1 across all gestational age groups (*P* < 0.001) with a particularly significant reduction observed among infants born at ≥26 weeks of gestation (Supplementary Figure 1).

### 3.1 Baseline characteristics

 Table 1 shows the perinatal characteristics of the 485 enrolled infants. Gestational age, birth weight, and sex distribution were comparable between Periods 1 and 2. However, multiple pregnancies were more frequent in Period 2, whereas the 1-min Apgar score was significantly lower in Period 1 (both *P* < 0.001). Pathologic chorioamnionitis and PROM ≥24 h were significantly more common in Period 1 than in Period 2 (*P* < 0.001 and *P* = 0.047, respectively).

**Table 1:** Baseline characteristics.

Characteristics	All preterm infants			Early preterm infants (GA 25–27 weeks)			Very preterm infants (GA 28–29 weeks)		
	Period 1 (*n* = 256)	Period 2 (*n* = 229)	*P*-value	Period 1 (*n* = 157)	Period 2 (*n* = 129)	*P*-value	Period 1 (*n* = 99)	Period 2 (*n* = 100)	*P*-value
GA, weeks	27.3 ± 1.4	27.5 ± 1.5	0.209	26.4 ± 0.8	26.4 ± 0.8	0.702	28.9 ± 0.6	28.9 ± 0.6	0.619
Birth weight, g	961.7 ± 245.7	939.5 ± 267.5	0.340	863.3 ± 178.7	826.6 ± 210.2	0.112	1,117.9 ± 257.1	1,085.1 ± 263.8	0.376
Male	128 (50.0)	121 (52.8)	0.594	83 (52.9)	68 (52.7)	1.000	45 (45.5)	53 (53.0)	0.356
Apgar score, 1 min	5.2 ± 1.8	5.9 ± 1.7	<0.001*	4.8 ± 1.7	5.4 ± 1.7	0.003*	5.9 ± 1.8	6.5 ± 1.5	0.006*
Apgar score, 5 min	7.7 ± 1.3	7.9 ± 1.6	0.232	7.4 ± 1.3	7.6 ± 1.7	0.466	8.1 ± 1.1	8.2 ± 1.5	0.521
SGA	43 (16.8)	52 (22.7)	0.128	22 (14.0)	30 (23.3)	0.063	21 (21.2)	22 (22.0)	1.000
Cesarean section	204 (79.7)	189 (82.5)	0.495	122 (77.7)	112 (86.8)	0.067	82 (82.8)	77 (77.0)	0.396
Multiple pregnancy	70 (27.3)	89 (38.9)	0.009*	48 (30.6)	50 (38.8)	0.185	22 (22.2)	39 (39.0)	0.016*ㅊ
Completion of antenatal steroid	139 (54.3)	125 (54.6)	1.000	89 (56.7)	72 (55.8)	0.977	50 (50.5)	53 (53.0)	0.833
Maternal DM	23 (9.0)	23 (10.0)	0.809	13 (8.3)	10 (7.8)	1.000	10 (10.1)	13 (13.0)	0.676
Maternal hypertension	45 (17.6)	52 (22.7)	0.195	26 (16.6)	24 (18.6)	0.767	19 (19.2)	28 (28.0)	0.195
Pathologic chorioamnionitis	134 (52.3)	90 (40.2)	0.010*	93 (59.2)	50 (38.8)	<0.001*	41 (41.4)	42 (42.0)	1.000
PROM (≥24 h)	81 (31.6)	53 (23.1)	0.047*	51 (32.5)	28 (21.7)	0.058	30 (30.3)	25 (25.0)	0.498
Oligohydramnios	67 (26.2)	47 (20.5)	0.175	44 (28.0)	30 (23.3)	0.435	23 (23.2)	17 (17.0)	0.358

GA, gestational age; SGA, small for gestational age; DM, diabetes mellitus; PROM, premature rupture of membranes. Values are presented as mean ± standard deviation (range) or *n* (%).

* *P* < 0.05.

In subgroup analyses, the 1-min Apgar score was significantly lower in Period 1 in both the EPI and VPI groups. Pathological chorioamnionitis was also more frequent in the EPI group during Period 1, whereas multiple pregnancies were more common in the VPI group during Period 2 (Table 1).

### 3.2 Neonatal outcome

Multivariate logistic regression analysis was adjusted for 1-min Apgar score, multiple pregnancies, pathologic chorioamnionitis, and PROM ≥ 24 h with gestational age and birth weight additionally included as clinically relevant variables regardless of univariate significance.

The rates of intubation in the delivery room and invasive surfactant administration were significantly lower in Period 2 (aOR: 0.20 and 0.09, respectively; *P* < 0.001 for both). In Period 2, CPAP failure occurred in 22.1% of infants within 48 h and in 35.3% at any time during hospitalization. Postnatal steroid (dexamethasone) use for BPD prevention was also significantly lower in Period 2 (aOR: 0.12, *P* < 0.001). Other short- and long-term outcomes did not significantly differ between the two periods (Table 2).

**Table 2:** Mortality and neonatal morbidity outcomes.

Characteristics	Period 1 (*n* = 256)	Period 2 (*n* = 229)	*P*-value	aOR^ab^ (95% CI)
Short term outcome				
Intubation at DR	197 (77.0)	105 (45.9)	<0.001*	0.20 (0.11–0.34)
CPAP failure (<48 h)		15/68 (22.1)		
CPAP failure (Anytime)		24/68 (35.3)		
Surfactant administration	228 (89.1)	208 (90.8)	0.081	1.86 (0.93–3.75)
Invasive surfactant administration	228 (89.1)	113 (49.3)	<0.001*	0.09 (0.05–0.16)
Noninvasive surfactant administration	0 (0.0)	97 (42.4)	0.984	N/A
Air leak	24 (9.4)	10 (4.4)	0.192	0.57 (0.25–1.32)
Pulmonary hemorrhage	7 (2.7)	2 (0.9)	0.055	0.20 (0.04–1.03)
Long term outcome				
BPD ≥ Grade 2	36 (14.1)	28 (12.2)	0.771	0.92 (0.51–1.64)
BPD ≥ Grade 2 or death	57 (22.3)	44 (19.2)	0.956	0.99 (0.60–1.63)
Late PHT (>1 month)	19 (7.4)	17 (7.4)	0.778	0.90 (0.44–1.84)
Duration of MV	18.8 ± 41.5	19.2 ± 38.9	0.591	1.95 (−5.18–9.09)
Duration of NIV	24.2 ± 29.6	23.8 ± 19.6	0.627	−1.08 (−5.45–3.29)
PNS use for BPD prevention (dexamethasone)	128 (50.0)	35 (15.3)	<0.001*	0.12 (0.07–0.21)
Other neonatal outcome				
IVH ≥ Grade 3	19 (7.4)	13 (5.7)	0.852	0.93 (0.43–2.02)
ROP with treatment	28 (10.9)	19 (8.3)	0.120	0.57 (0.28–1.16)
NEC ≥ Stage 2	27 (10.5)	22 (9.6)	0.728	1.12 (0.60–2.09)
Culture proven sepsis	33 (12.9)	24 (10.5)	0.604	0.86 (0.48–1.54)
Mortality	31 (12.1)	21 (9.2)	0.676	0.87 (0.45–1.67)

DR, delivery room; BPD, bronchopulmonary dysplasia; PHT, pulmonary hypertension; MV, mechanical ventilation; NIV, noninvasive ventilation; PNS, postnatal steroid; IVH, intraventricular hemorrhage; ROP, retinopathy of prematurity; NEC, necrotizing enterocolitis. AOR, adjusted odds ratio; CI, confidence interval. Values are presented as *n* (%). BPD severity was graded according to the Jensen criteria.

a Adjusted for gestational age, birth weight, Apgar score at 1 min, multiple pregnancies, pathologic chorioamnionitis, and PROM ≥24 h. Values are adjust odds ratio (aOR) for binary outcomes and adjust regression coefficients (*β*) for continuous outcomes. .

b Period 1 (reference category) vs. Period 2.

*P*-values were derived from univariate comparisons (chi-square or *t* test) between Periods 1 and 2.

* *P* < 0.05.

Table 3 presents the comparison of outcomes between Periods 1 and 2 by gestational age subgroups. In Period 2, delivery room intubation, invasive surfactant administration, and postnatal steroid use for BPD prevention significantly decreased. In addition, among EPI, the incidence of ROP requiring treatment was significantly reduced (aOR: 0.47; *P* = 0.048) and CPAP failure was also observed.

**Table 3:** Mortality and neonatal morbidities in the EPI and VPI groups.

Characteristics	Extremely preterm infants (GA 25–27 weeks)				Very preterm infants (GA 28–29 weeks)			
	Period 1 (*n* = 157)	Period 2 (*n* = 129)	*P*-value	aOR^a^^,^^c^ (95% CI)	Period 1 (*n* = 99)	Period 2 (*n* = 100)	*P*-value	aOR^b^^,^^c^ (95% CI)
Short term outcome								
Intubation at DR	135 (86.0)	83 (64.3)	0.004*	0.26 (0.12–0.55)	62 (62.6)	22 (22.0)	<0.001*	0.12 (0.05–0.27)
CPAP failure (<48-Hour)	—	9/31 (29.0)			—	6/37 (16.2)		
CPAP failure (Anytime)	—	15/31 (48.4)			—	9/37 (24.3)		
Surfactant administration	148 (94.3)	125 (96.9)	0.097	3.44 (0.80–14.80)	80 (80.8)	83 (83.0)	0.576	1.26 (0.56–2.82)
Invasive surfactant administration	148 (94.3)	84 (65.1)	<0.001*	0.09 (0.04–0.22)	80 (80.8)	29 (29.0)	<0.001*	0.08 (0.04–0.18)
Noninvasive surfactant administration	0 (0.0)	42 (32.6)	0.988	N/A	0 (0.0)	55 (55.0)	0.985	N/A
Air leak	21 (13.4)	6 (4.7)	0.048*	0.37 (0.14–0.99)	3 (3.0)	4 (4.0)	0.314	2.44 (0.43–13.89)
Pulmonary hemorrhage	5 (3.2)	0 (0.0)	0.994	N/A	3 (3.0)	5 (5.0)	0.521	0.50 (0.06–4.18)
Long term outcome								
BPD ≥ Grade 2	32 (20.4)	23 (17.8)	0.522	0.81 (0.42–1.55)	4 (4.0)	5 (5.0)	0.641	1.43 (0.32–6.33)
BPD ≥ Grade 2 or death	48 (30.6)	34 (26.4)	0.653	0.88 (0.49–1.56)	9 (9.1)	10 (10.0)	0.673	1.26 (0.43–3.68)
Late PHT (>1mo)	16 (10.2)	13 (10.1)	0.620	0.81 (0.35–1.86)	3 (3.0)	4 (4.0)	0.838	1.18 (0.24–5.94)
Duration of MV	27.9 ± 50.6	26.1 ± 32.2	0.653	−2.38 (−12.76–8.01)	4.5 ± 8.5	10.3 ± 44.7	0.109	7.58 (−1.71–16.89)
Duration of NIV	73.5 ± 71.2	73.6 ± 50.0	0.166	−4.86 (−11.73–2.02)	19.8 ± 26.2	37.8 ± 48.1	0.008*	5.79 (1.51–10.01)
PNS use for BPD prevention (Dexamethasone)	110 (70.1)	31 (24.0)	<0.001*	0.11 (0.06–0.21)	18 (18.2)	4 (4.0)	0.005*	0.17 (0.05–0.58)
Other neonatal outcome								
IVH ≥ Grade 3	15 (9.6)	9 (7.0)	0.808	0.89 (0.35–2.27)	4 (4.0)	4 (4.0)	0.933	0.94 (0.21–4.25)
ROP with treatment	28 (17.8)	17 (13.2)	0.048*	0.47 (0.22–0.99)	0 (0.0)	2 (2.0)	0.998	N/A
NEC ≥ Stage 2	23 (14.6)	17 (13.2)	0.854	0.94 (0.46–1.91)	4 (4.0)	5 (5.0)	0.483	1.65 (0.41–6.63)
Culture proven sepsis	27 (17.2)	15 (11.6)	0.291	0.68 (0.33–1.39)	6 (6.1)	9 (9.0)	0.568	1.39 (0.45–4.26)
Mortality	26 (16.6)	15 (11.6)	0.447	0.75 (0.35–1.58)	5 (5.1)	6 (6.0)	0.706	1.30 (0.33–5.16)

NEC, necrotizing enterocolitis; ROP, retinopathy of prematurity; IVH, intraventricular hemorrhage; BPD, bronchopulmonary dysplasia; PTH, pulmonary hypertension. BPD severity was graded according to the Jensen criteria. Values are presented as *n* (%).

*P-*values were derived from univariate comparisons (chi-square or *t*-test) between periods 1 and 2.

a Adjusted for gestational age, birth weight, Apgar score at 1 min, and pathologic chorioamnionitis.

b Adjusted for gestational age, birth weight, Apgar score at 1 min, and multiple pregnancies. Values are adjust odds ratio (aOR) for binary outcomes and adjust regression coefficients (*β*) for continuous outcomes.

c Period 1 (reference category) vs. Period 2. AOR, adjusted odds ratio; CI, confidence interval.

* *P* < 0.05.

### 3.3 Risk factors for CPAP failure

Table 4 presents the perinatal characteristics of infants in Period 2 who were initially managed with CPAP, comparing between those with CPAP success and failure within 48 h and between those with CPAP success and failure at any time during hospitalization. In both comparisons, the completion rate of antenatal steroid therapy was significantly lower in the failure groups.

**Table 4:** Perinatal characteristics of infants with CPAP failure.

Characteristics	48 h			Anytime		
	CPAP failure (*n* = 15)	CPAP success (*n* = 53)	*P*-value	CPAP failure (*n* = 24)	CPAP success (*n* = 44)	*P*-value
GA, weeks	27.6 ± 1.3	28.0 ± 1.2	0.274	27.5 ± 1.4	28.2 ± 1.1	0.019*
Birth weight, g	896.3 ± 212.2	989.2 ± 247.3	0.093	856.7 ± 206.8	1,020.7 ± 244.8	< 0.001*
Male	5 (33.3)	26 (49.1)	0.432	9 (37.5)	22 (50.0)	0.463
Apgar score, 1 min	6.3 ± 1.4	6.5 ± 1.0	0.534	6.3 ± 1.3	6.5 ± 0.9	0.534
Apgar score, 5 min	8.2 ± 0.9	8.5 ± 1.4	0.505	8.2 ± 1.0	8.5 ± 1.4	0.489
SGA	5 (33.3)	12 (22.6)	0.612	8 (33.3)	9 (20.5)	0.379
Cesarean section	15 (100.0)	44 (83.0)	0.495	23 (95.8)	36 (81.8)	0.209
Multiple pregnancy	6 (40.0)	13 (24.5)	0.394	9 (37.5)	10 (22.7)	0.310
Completion of antenatal steroid	5 (33.3)	40 (75.5)	< 0.001*	8 (33.3)	37 (84.1)	< 0.001*
Maternal DM	0 (0.0)	5 (9.4)	0.499	0 (0.0)	5 (11.4)	0.219
Maternal hypertension	18 (34.0)	7 (46.7)	0.550	10 (41.7)	15 (34.1)	0.722
Pathologic chorioamnionitis	4 (26.7)	26 (49.1)	0.212	9 (37.5)	21 (47.7)	0.578
PROM (≥24 h)	0 (0.0)	13 (24.5)	0.078	0 (0.0)	13 (29.5)	0.008*
Oligohydramnios	0 (0.0)	8 (15.1)	0.251	0 (0.0)	8 (18.2)	0.067

GA, gestational age; SGA, small for gestational age; DM, diabetes mellitus; PROM, premature rupture of membranes.

Values are presented as means ± standard deviation (range) or *n* (%).

* *P* < 0.05.

Among infants who experienced CPAP failure at any time, gestational age and birth weight were significantly lower, while PROM was more frequent in the success group. In multivariate analysis, completion of antenatal steroid therapy was significantly associated with a lower risk of CPAP failure (Table 5).

**Table 5:** Regression analysis investigating risk factors for CPAP failure at any time.

Characteristics	CPAP failure (*n* = 24)	CPAP success (*n* = 44)	aOR^a^ (95% CI)	*P*-value
GA, weeks	27.5 ± 1.4	28.2 ± 1.1	0.88 (0.53–1.49)	0.642
Birth weight, g	856.7 ± 206.8	1,020.7 ± 244.8	1.00 (1.00–1.00)	0.287
Completion of antenatal steroid	8 (33.3)	37 (84.1)	0.17 (0.04–0.63)	0.008*
PROM (≥24 h)	0 (0.0)	13 (29.5)	0.00 (0.00-Inf)	0.992

GA, gestational age; PROM, premature membrane rupture; aOR, adjusted odds ratio; CI, confidence interval; CPAP, continuous positive airway pressure.

Values are presented as mean ± standard deviation (range) or *n* (%).

a CPAP fail (reference category) vs. CPAP success.

* *P* < 0.05.

### 3.4 Statistical process control chart for QI

**Figure 2:**
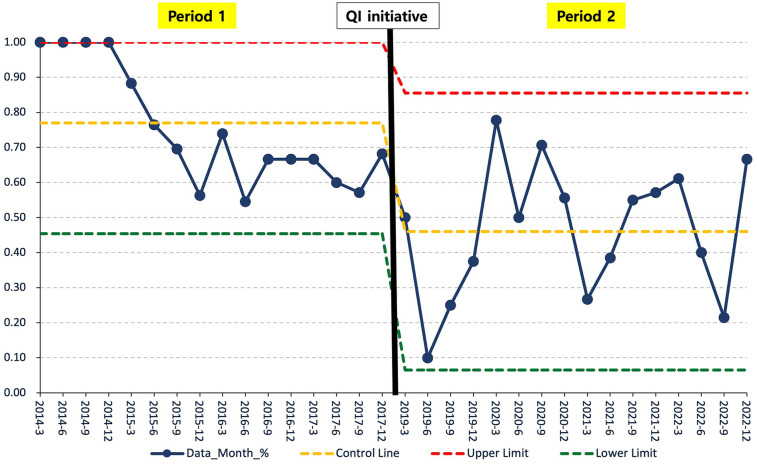
Statistical process control chart for QI: initial intubation rate in period 1 vs. Period 2. Statistical process control chart showing quarterly initial intubation rates in the delivery room from 2014 to 2022. A vertical reference line marks the transition point in 2018 by dividing the data into Periods 1 and 2. The control limits were used to detect statistically significant variations. SPC, statistical process control; QI, quality improvement.

Following the implementation of the QI initiative, a sustained downward trend was observed, indicating a shift to a new stable process. Control limits were applied to detect statistically significant variation, and the 2018 transition period was marked with a vertical reference line. The *X*-axis denotes calendar quarters (Q1–Q4), and the *Y*-axis denotes the delivery room intubation rate (Figure 2).

## 4 Discussion

The QI initiative aimed to enhance neonatal respiratory outcomes by promoting the CPAP use in delivery rooms. The intervention was associated with a reduction in the initial intubation rate and with lower postnatal steroid use for BPD prevention, suggesting the feasibility of selective intubation and proactive use of CPAP in preterm infants.

CPAP is beneficial for preterm infants experiencing respiratory distress after birth and may reduce adverse outcomes of VPIs compared with endotracheal intubation (11–13). It is a less invasive form of respiratory support compared with intubation or positive pressure ventilation (14–17). To optimize noninvasive respiratory support, appropriate techniques and effective devices are essential. However, due to challenges in maintaining adequate positive end-expiratory pressure from the delivery room to the NICU, routine endotracheal intubation is often performed (18–20). Historically, many preterm infants were intubated immediately after birth due to limited provider experience, difficulty sustaining noninvasive support during transport, and high CPAP failure rate (21).

In the COIN trial, preterm infants born between 25 and 28 weeks of gestation were randomized to early nasal CPAP or intubation group. Although there was no significant difference in BPD or mortality rates, the early CPAP group had reduced oxygen requirements at 28 days of life and a shorter ventilation duration, despite a higher pneumothorax rate (22). Similarly, the SUPPORT trial randomly assigned infants born between 24 + 0 and 27 + 6 weeks of gestation to either tracheal intubation with surfactant treatment within the first hour of life or to CPAP initiation in the delivery room. The early CPAP application strategy used in this trial was associated with a lower intubation rate in the delivery room, reduced duration of mechanical ventilation, and decreased use of postnatal corticosteroids (14). Additionally, in a study by Shukla et al., noninvasive ventilation was applied to preterm infants born at less than 24 weeks of gestation. While this approach did not reduce the risk of BPD or mortality compared to intubation with early surfactant application, it demonstrated feasibility in this population (23). In our study, CPAP failure occurred in approximately one-third of infants, consistent with previous reports (1, 24).

Our study differs from previous research in that it observes a notable transformation spanning almost a decade. This transformation was driven by the introduction of a QI initiative.

In this study, we found that the adoption of active initial CPAP usage, as opposed to routine intubation, was associated with lower postnatal steroids use for BPD prevention and demonstrated safety and feasibility, even in EPI. Although the incidence of BPD was not significantly reduced, the reduction in postnatal steroid use suggests the feasibility of potential long-term improvement in neurodevelopmental outcomes and a lower risk of steroid-related adverse effects, including growth restriction, hyperglycemia, and infection (25, 26). The reduction in invasive ventilation likely contributed to decreased steroid use for BPD prevention and extubation support. Despite a lower intubation rate, BPD incidence remained unchanged, suggesting that noninvasive strategies may be safe alternatives without compromising respiratory outcomes.

This study has some limitations. This single-center, retrospective observational study included a limited number of infants. While gestational age, birth weight, and other demographic factors were statistically adjusted, the observed improvement in 1-min Apgar scores during Period 2 may reflect enhanced overall care quality from a QI perspective. However, the lack of evaluation of postnatal steroid use on neurodevelopmental outcomes and the absence of long-term follow-ups, such as assessments using the Bayley Scale, limited our ability to fully understand the long-term implications of the interventions. In addition, differences between the two periods may not be solely due to the QI initiative, as changes in delivery room practices and NICU staffing could also have influenced to the outcomes.

In conclusion, the selective use of portable CPAP was associated with a marked reduction in initial intubation rates and decreased use of postnatal steroids for BPD prevention. Although no significant differences were observed in other morbidities, there was a trend toward lower BPD incidence, especially in EPI. These findings support the use of CPAP as a safe and effective alternative to routine intubation in preterm infants not requiring invasive respiratory support.

## Data Availability

The data analyzed in this study is subject to the following licenses/restrictions: the datasets generated and/or analyzed during the current study are not publicly available due to institutional restrictions but are available from the corresponding author upon reasonable request. Requests to access these datasets should be directed to Se In Sung, sein.sung@samsung.com.
