# Supplementary material for: Increasing continuous positive airway pressure use rates in the delivery room for very preterm infants: a quality improvement initiative
Source: Front Pediatr. 2025 Nov 6;13:1697565. doi: 10.3389/fped.2025.1697565 (PMC12631424; doi:10.3389/fped.2025.1697565)
Supplement: Supplementary file 1 [file Supplementaryfile1.docx]

Supplementary Material

# Supplementary Figures


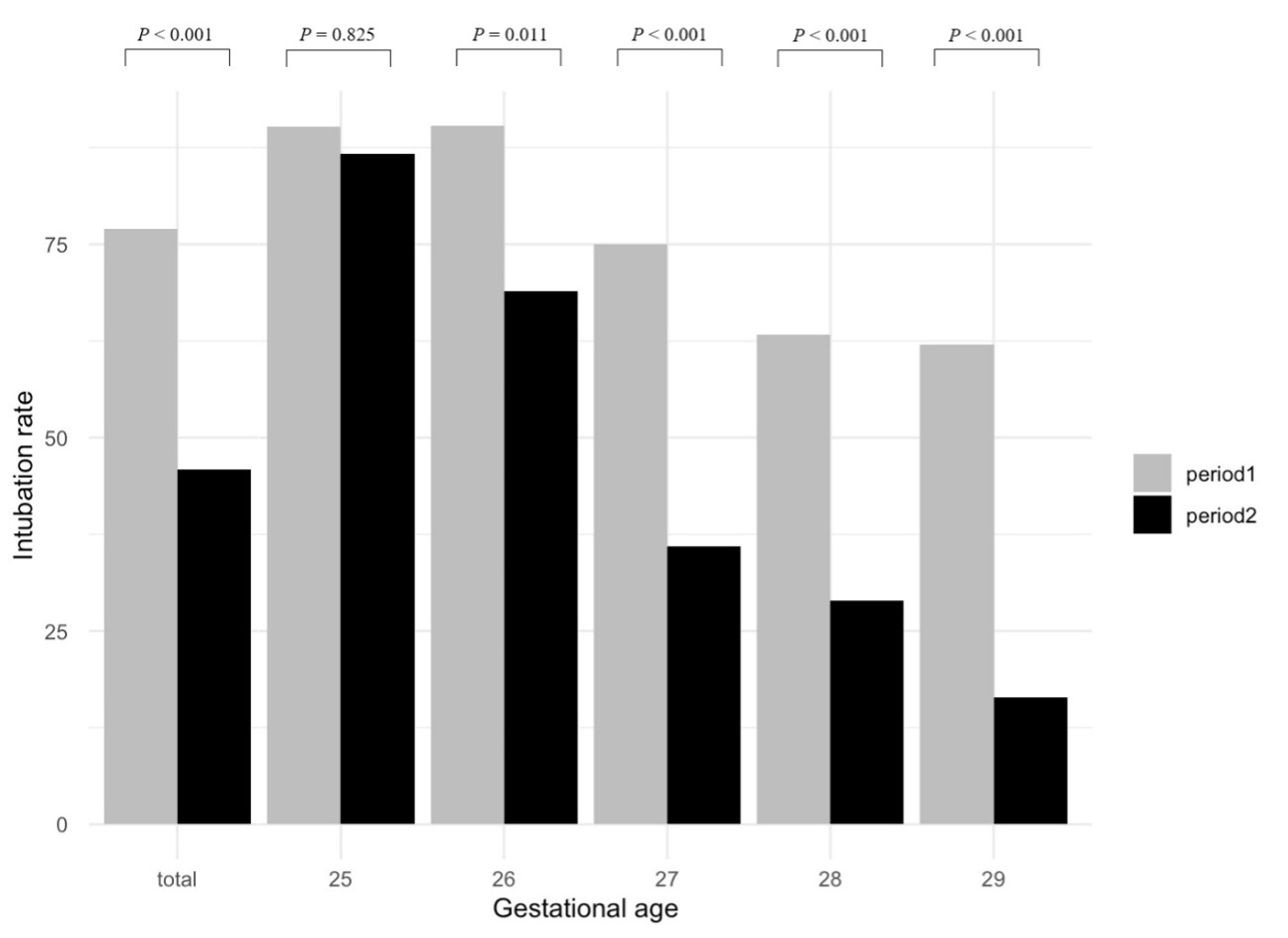


**Supplementary Figure 1.** Initial intubation rates stratified by gestational age groups in Period 1 and Period 2. Overall, the rates were consistently lower in Period 2 compared with Period 1, with a particularly significant reduction observed among infants born at ≥26 weeks of gestation.
